# Dictyostelium: The Mathematician’s Organism

**DOI:** 10.2174/13892029113149990010

**Published:** 2013-09

**Authors:** A.J Durston

**Affiliations:** IBL, Sylvius Laboratory, Wassenaarseweg 72, 2333 BE, Leiden, The Netherlands

**Keywords:** Dictyostelium, Excitable medium, Waves, Spirals, Dislocation, Somitogenesis.

## Abstract

This article was to have been written by Kees Weijer, an outstanding pioneer in *Dictyostelium* research. It was (and is) to celebrate J.T. Bonner’s and Weijer’s contributions to the field and those of the other great pioneers. Unfortunately, Weijer was unable to write his article, due to ill health and since I have some knowledge of this field, I took it over. The article summarises some main results and ideas in Dictyostelium research and their relevance for development of more advanced organisms.

## 1 DICTYOSTELIUM DISCOIDEUM: INTRODUCTION

**Fig. (1):**
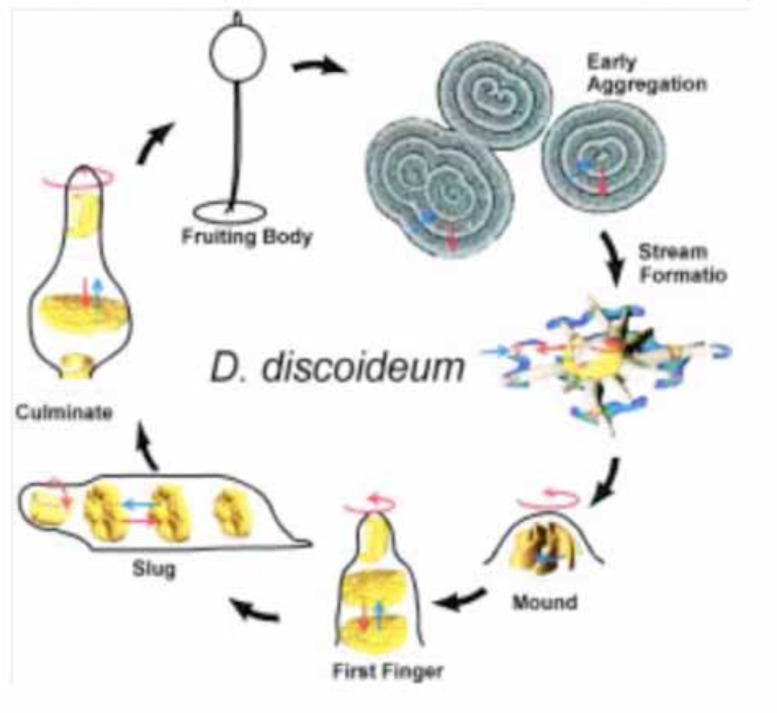
The life cycle of *Dictyostelium* discoideum. Single *Dictyostelium* ameobae aggregate via relayed waves of movement due to chemotaxis and relaying. The aggregating cells collect into streams and then into mounds. Each mound makes one or more nipple like tips which oranise finger shaped masses. THGe masses fall over and migrate (slugs). After a varying period of migration, each slug rounds up and makes a sporangium like fruiting body, The fruiting body consists of a stalk and basal disc of dead stalk cells and iving spores, each of which can hatch to release an amoeba.

Slime mould! That sounds like something you might find at the bottom of your fridge after you have been away for the summer. In fact, the cellular slime mould *Dictyostelium* is a very simple multicellular organism that has been used as a paradigm for understanding multicellular development. Its multicellular organisation is mediated by a stunningly beautiful mechanism (below) and is so simple and well understood it is almost mathematically defined: and has attracted many mathematicians, physicists and computer scientists to biology. At one stage in its life cycle (Fig. **1**), *Dictyostelium* is a population of independent feeding cells that phagocytose bacteria. At another, starvation causes this organism to become multicellular. The cells aggregate by chemotaxis towards an attractant, acrasin, that has been identified for *Dictyostelium* discoideum as cyclic AMP. They make multicellular mounds that develop a distinct central tip. The mound grows up into a finger like structure, which contains at least two cell types: anterior prestalk cells and posterior prespore cells. The prestalk-prespore pattern is quantitatively defined and is regulative. In *Dictyostelium* discoideum, the finger falls over and migrates as the so-called slug stage. If a slug is cut up, each piece regulates back to a slug with a normal pattern. Its pattern is thus regulated by feedback mechanisms. The slug finally stops migrating and constructs a simple fruiting body. The elongated slug rounds up to an inverted mushroom shape (Mexican Hat) and the organism’s tip begins to extend again. This time as the tip of an elongating stalk, consisting of an extracellular sheath containg dead vacuolated stalk cells, which is pushed down through the cell mass. The prestalk cells progressively enter the stalk from the tip, vacuolate and die. The prespore cells are carried aloft and progressively undergo final differentiation to immobile encapsulated spore cells. These processes generate a mould sporangium like fruiting body (sorocarp). A subpopulation of prestalk like cells remain at the base of the stalk and vacuolate and die to make a basal disc at the bottom of the stalk. 

## 2 THE PIONEERS

This whole process has been investigated by a number of wonderful pioneers, who worked out the phenomenology of *Dictyostelium* development, its genetics, molecular biology, DNA sequence, cell biology etc. The two original pioneers were Kenneth Raper, who discovered *Dictyostelium* discoideum in Camel dung from the Bronx zoo and John Bonner, the pioneer per excellence, who has inspired the field and has presumably had a different original idea for each and every day of his life. Endorsement of Bonner’s latest book (Bonner, 2008): "Few scientists or authors can claim that the analyses and insights in their latest book are based on sixty years of original research, exploration, and childlike enthusiasm. We should be enormously grateful that John Tyler Bonner could make that claim about the career he has spent with cellular slime molds. His book is beautifully written, enlightening, fascinating, historical yet up-to-date, whimsical when appropriate, and informative throughout in its analysis of two of evolution's major themes--multicellular organization and sociality."--Brian Hall, coauthor of *Strickberger's Evolution. *A very important discovery however was made by a little known Englishman, Brian Shaffer (Shaffer, 1975) who realised that the waves of movement seen in early *Dictyostelium* aggregation movies made by John Bonner must mean that *Dictyostelium* cells are excitable. That when a *Dictyostelium* cell receives a chemotactic signal, it also emits one. Shaffer showed that if aggregation stage *Dictyostelium* discoideum cells are pulsed with Bonner’s chemoattractant cyclic AMP, they secrete cyclic AMP in response themselves. This is the relay mechanism. For simplicity, I am going to concentrate here only on the cell biology of movement coordination via chemotaxis and signal relay and ignore the other important aspects including all of the molecular biology.

## 3 HISTORICAL: TILL 1979

Shaffer’s discovery showed that an important property of *Dictyostelium* is that it consists of a population of excitable cells. A field of aggregating *Dictyostelium* discoideum cells is an isotropic excitable medium (Durston, 1973). It shows waves of chemotactic cell movement, reflecting waves of secreted C-AMP. Waves are unidirectional because, once a *Dictyostelium* cell has signalled, it enters a refractory priod- a so called adapted state. So when two waves meet they annihilate each other. Centres compete and the highest frequency centres win. There are two geometrical forms of waves in a (2D) aggregation field, 1 cell layer thick. Concentric rings are initiated early on from centres that presumably contain cells capable of autonomously emitting a periodic signal. These early concentric centres emit periodic waves with a 5’ period or a multiple of this value. Presumably a periodic centre with a 5’ signal is gated by the time dependent refractory period of the responding cells (Durston 1974). A second wave form that arises during the course of aggregation is a self sustaining involute Archimedean spiral. A continuous wave front spirals around and out out from a central core, generating a spiral with parallel coils. Spirals have a time dependent period, that starts long (up to around 10 minutes) and finally stabilses at a short time interval (about 2 1/2 min). There is reason to think that spirals gravitate to the refractory period of the aggregating cells, which is known to decrease with time. Because spirals have a high frequency, they out compete early concentric centres and a *Dictyostelium* aggregation field comes to be dominated by spirals provided the cells are highly excitable (Durston, 1973). That is until the aggregates form tips. See below. These properties of *Dictyostelium* aggregation fields resemble those of other excitable media, like Zhabotinsky reagent, the oscillating chemical reagent studied by A.T. Winfree (Winfree 1972, 1973,), and others. Zhabotinsky reagent shows clearly how spirals can be formed. If you perturb the liquid reagent, waves break and the excitable wavefronts curl around behind the refractory zones at their broken ends. Each pair of broken wave ends becomes a pair of counter-rotating spirals (Winfree 1972). In *Dictyostelium*, we can assume that such spirals are initiated by inhomogeneities in the aggregation field that break waves (Durston, 1973). These properties have made *Dictyostelium* a beloved object of study for mathematicians, theoretical physicists, computer modellers and others who appreciate its elegant and simple morphogenesis. It has brought many, including such eminent scientists, from other backgrounds to biological research. In the following sections, we will concentrate only on the importance of this chemotaxis-relay system for multicellular organisation, while ignoring many other important aspects, like the molecular mechanisms of chemotaxis and relay, pattern formation, molecular genetics, etc., etc..

## 4 WAVES IN DEVELOPMENT

**Fig. (2):**
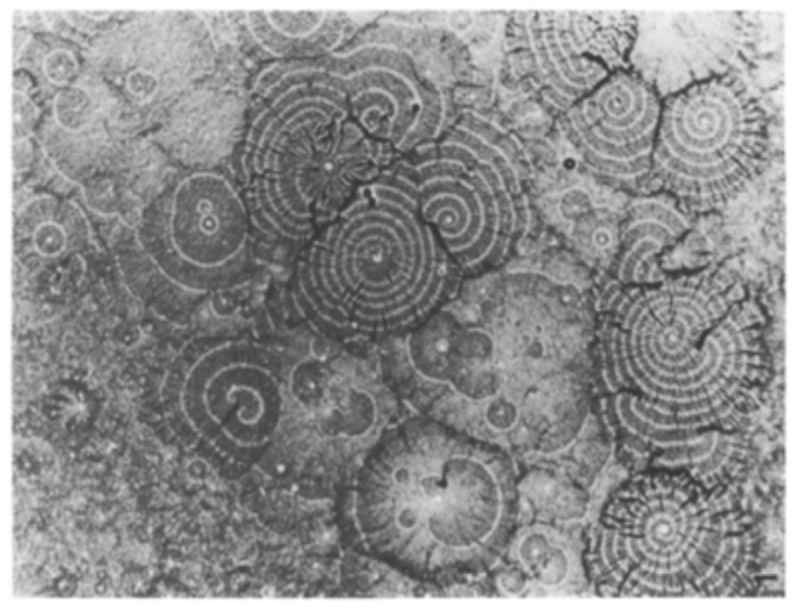
Waveforms in Dictyostelium .. Spiral and concentric waves in a Dictyostelium aggregation field. The spirals are high frequency, leading to closely packed waves. The concentrics are lower frequency.

There is evidence that the chemotaxis-relay system that regulates *Dictyostelium* aggregation also regulates its later multicellular morphogenesis. Erecting *Dictyostelium* sorocarps are typified by periodic pulsatile movements during culmination (Durston *et al.*, 1976). All later stages actually show periodic waves of movement, like aggregates. These are seen in late aggregates, slugs and erecting sorocaps (ie in all stages) (Durston *et al.* 1978,1979). An idea of the types of waves we should expect in these 3 dimensional structures has been predicted in Zhabotinsky reagent, where it has been shown that 2D spirals are actually 3D scroll waves in three dimensions (Winfree 1973) (Fig. **2**). It has been shown that such scroll waves commonly occur in closed torus rings, where the axis of the torus often has a twist (Barkley *et al.*, 1987). In *Dictyostelium*, there were many indications that 3D spirals ought to be involved in later morhogenesis. *Dictyostelium* slug migration is helical with the tip making a corkscrewing motion (D.Drage, pers. Comm.). All *Dictyostelium* later structures are based on a cylindrical format, which could naturally be organised by a 3D spiral (scroll) wave. Late aggregates, which can stop development at this stage without forming a tip, often become doughnuts, where cells migrate over the top inward or over the top outward (Durston 1978, 1999). These aggregates are evidently organised by the two counterrotating (clockwise/ anticlockwise) forms of a torus scroll wave. The aggregate shown evidently also has a twist in its axis. Some late aggregates form short sausages, where the visible upper cells rotate clockwise or anticlockwise over the sausage. These presumably represent linear scroll waves. Culminating slugs, which round up always contain a 3D spiral at the beginning of culmination (Durston *et al.*, 1979). Most attention however has focussed on the slug stage because this is embryo- like and forms a regulative axial pattern. We will restrict our attention here to this stage and to aggregation. We examined 56 migrating *Dictyostelium* slugs for their movement patterns and detected forward cell movement flows in the prespore zones of 46. We only detected 3D scroll spirals in 3 of them (in their prestalk zones) although our main purpose in initiating this study was to look for 3D spirals in slugs. We therefore dismissed this waveform, thereby totally missing the point and should have looked harder.

## 5 WEIJER’S CONTRIBUTION

Kees Weijer’s contribution to developmental biology in general and *Dictyostelium* science in particular should not be underestimated. He has made important advances in; bioimaging; mathematical modelling; in the molecular genetics of chemotaxis and oscillatory signalling; in the phenomenology of *Dictyostelium* wave propagation; and demonstration and characterisation of chemotaxis in a vertebrate embryo (chick). All of this has been published in many scientific papers, some of which are highly cited. Weijer’s greatest achievement however was to identify a possible solution to *Dictyostelium* multicellular organisation in the slug. The solution to *Dictyostelium* multicellularity was staring us in the face when I left the field around 1980. We had seen that *Dictyostelium* multicellular stages are characterised by periodic movements and periodic waves of movement. We had seen that prestalk and prespore cells, which differentiate from different cell cycle phases during starvation (Mac-Donald and Durston, 1984, Weijer *et al.*, 1984a) sort out chemotactically (Matsukuma and Durston, 1979). This sorting was clearly part of, though presumably not all of, the prestalk/prespore patterning process. Clearly, the chemotaxis relay system is important for later development. Siegert and Weijer, (1992) saw that the prestalk zone of *Dictyostelium* slugs always contains a scroll wave, while the prespore zone always has backward propagating plane waves as we and he saw (Durston *et al.*, 1979, Siegert and Weijer, 1992). Scientific breakthroughs almost always consist of small steps. Weijer saw what we all missed. He saw what Archimedes saw before him: that a scroll can only do useful work, so it can control the forward movement of slugs or cell sorting, if you put a twist in its axis. Rotational energy is thus converted to linear energy. The Archimedean spiral has to become an Archimedes screw. This principle underlies many important engineering inventions, from hydroelectric power stations to jet turbines. Weijer’s group have detected twisted scroll waves in *Dictyostelium* *mucoroides*. They have modelled slug movement and scroll waves in the *Dictyostelium* slug and can produce stable twisted scroll waves in their models under appropriate conditions (Bretschneider *et al.*, 1995, 1999, Vasiev and Weijer, 1999,. 2003, Weijer, 1999) An important example is when conditions in the prespore zone of the slug are such as to produce a frequency gradient within this zone. 

## 6 FURTHER THOUGHTS

**Fig. (3):**
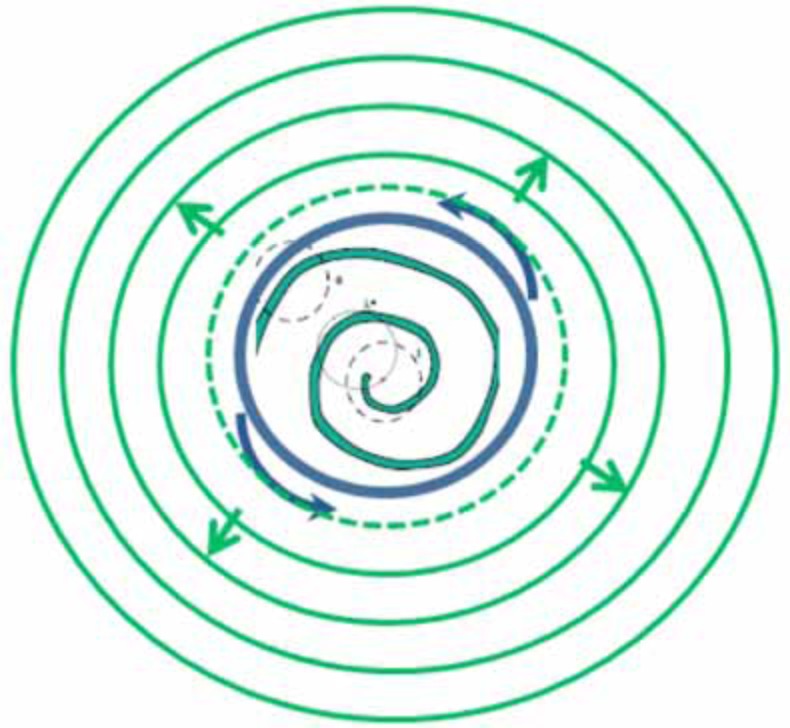
Dislocation. A very small aggregate of prestalk cells (outlined by the blue ring) forms an early stage tip at the late aggregation-mound stage in *Dictyostelium*. This aggregate contains a spiral wave (green spiral line). It counter-rotates, due to its movent reponse to the spiral wave. This small aggregate is seen by the remaining cells in the mound (a mixture of prespore and prestalk cells) as an integral continuous signal source. It therefore acts effectively as a point source, initiating high frequency concentric target waves of relaying in the mound. What is seen is that a small rotating aggregate initiates the tip and that this evidently spiral aggregate none the less initiates concentric high frequency waves of relaying. It is also known that an artificial continuous source of C-AMP initiates concentric high frequency waves of relaying.

The *Dictyostelium* mechanism may be more radical than Weijer and colleagues realised. During late aggregation, the slime mould cells differentiate to two cell types: the prestalk and prespore cells. These cell types originate from cells that were in different phases of the cell cycle at the moment of starvation (MacDonald and Durston, 1984). They sort out chemotactically during late aggregation, so that prestalk cells come to be in the tip Matsukuma and Durston, 1979). They have a difference in excitability properties: namely a difference in their signalling frequency (Weijer *et al.*, 1984b). These differences may lead to interesting movement and wave phenomena. It is possible that the prestalk cell mass in the tip and the remaining cell mass in the late aggregate, move independently. We have observed that the aggregate tip, which clearly contains a scroll wave can, at least under certain conditions, initiate concentric waves in the remaining cell mass. This could potentially be accounted for by Weijer’s twisted scroll mechanism but we also think it possible that there is dislocation between wave propagation in the prespore and prestalk parts of the *Dictyostelium* cell mass (Fig. **3**). We note that there is no sign of a scroll wave outside the tip and that this situation arises in originally concentric as well as originally spiral aggregates. These thoughts are discussed in detail elsewhere (Durston, in prep.).

## 7 GENERAL IMPLICATIONS

**Fig. (4):**
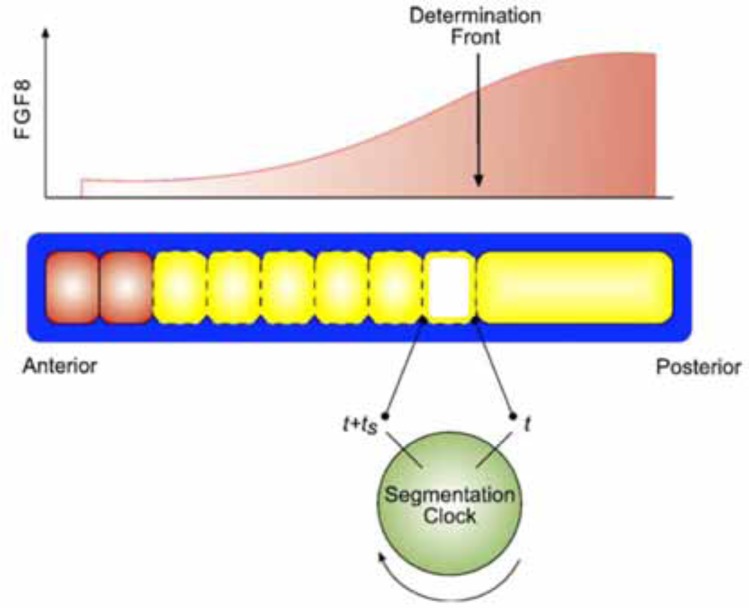
Oscillators, and wave propagation in higher organisms. The somitogenesis clock. A temporally oscillating system of gene expression (somitogenesis clock) generates periodic propagating waves that interact with a wave of fixation activity to generate spatially periodic somites (Baker and Maini, 2012).

Has this work in *Dictyostelium* just been a training exercise or does it have general implications? The jury is still out on that one. Work was initiated with *Dictyostelium* because the pioneers thought that this would prove a suitable model system for planning cell biological studies of metazoan embryogenesis. In fact, the evolutionary distance between *Dictyostelium* and the metazoa is enormous. None of the interesting developmental genes in metazoa are found in *Dictyostelium*- eg the metazoan Hox genes are not. Many including myself have looked to see if chemotaxis and signal relay are important in metazoan embryos. The early results were not encouraging. Gingle *et al.*, 1972, claimed a movement response of the early chick embryo to a localised source of C-AMP. This seems an unlikely chemoattractant. These results have not been repeated or expanded. Nanjudiah (1972) showed that explanted organisers from early Axolotl embryos served uniquely as sources of attractant for *Dictyostelium* ameobae, suggesting that these secrete c-AMP. Stern and Goodwin (1972) showed early periodic movements in the chicken embryo. This is an interesting result which is presumably worthy of further investigation. An early model (Goodwin and Cohen, 1972) proposed that different waves of excitation initiated by the vertebrate organiser, could interact to generate a size independent (scaled) pattern. This recalls the situation with the somitogenesis clock (below). Recently, after elucidation of molecular genetic mechanisms inMetazoa, things are more hopeful (Fig. **4**). Several people but notably Kees Weijer, have discovered that chemotaxis is important during metazoan development. Weijer’s results are spectacular. He showed that at least 4 different signalling pathways are involved in setting up the main body axis in the chicken gastrula. Both positive and negative chemotaxis are involved but no sign of signal relay was detected. See this hot topic, part 1, June 2012: article by Chuai *et al.* (2012). Coupled oscillators and wave propagation have recently been shown to be important in metazoan development in a small number of mechanisms. The best characterised of these is the ‘somitogenesis clock’ This is an oscillating system of gene expression that generates the embryo’s periodic somites (mesodermal segments) (Baker *et al.*, 2012). It also controls the embryo’s axial Hox pattern of positional information (Peres *et al.*, 2006, Durston, 2011). Both of these functions involve ‘time space translation’. This oscillating system is actually a wave generator, like a *Dictyostelium* cell mass. Because of its wave properties, it manifests ‘scaling’; size independence of its axial pattern, which is regulated via a size independent pphase gradient (Lauschke *et al.*, 2013). It may control other important properties. Regardless of any evolutionary connections, this wave propapgation system will obey certain rules that predict how it will behave. We have insight into these rules from the simple *Dictyostelium* system. A question that arises is why *Dictyostelium* development seems so incredibly elegant compared, for example, to characterising extracellular matrix in a mouse embryo (although there have been some very elegant discoveries in metazoan development). *Dictyostelium* development is indeed very elegant. It is elegant because it is very simple. So simple that it is now very well understood and it has been possible to characterise the key mechanisms very exactly- to the extent that mathematical models are now realistic. Mathematicians and physicists have been inspired by this elegance and simplicity to move to biological research. Metazoan developmental mechanisms are clearly much more complex as demonstrated by comparing Weijer’s triple positive and negative chemotactic system in the chicken gastrula with the single positive chemotactic system in *Dictyostelium*. When we get to know them properly, they will assuredly be just as beautiful.

## References

[1] Baker RE, Schnell S, Maini PK (2006). A clock and wavefront mechanism for somite formation. Dev. Biol.

[2] Barkley D, John R, Jack S (1987). Turner Observations of a torus in a model of the Belousov–Zhabotinskii reaction. J. Chem. Phys.

[3] Bonnerm JT (2008). The Social Amoebae: The Biology of Cellular Slime
Molds.

[4] Manli C, David H, Cornelis JW (2012). Collective Epithelial and Mesenchymal Cell Migration During Gastrulation. Curr. Genomics.

[5] Dormann D, Weijer CJ (2001). Propagating chemoattractant waves
coordinate periodic cell movement in Dictyostelium slugs. Development.

[6] Dormann D, Weijer C, and Siegert F (1997). Twisted scroll waves organize
*Dictyostelium* mucoroides slugs. J. Cell. Sci.

[7] Durston A (1973). Dictyostelium discoideum aggregation fields as excitable media. J. Theor. Biol.

[8] Durston AJ (1974). Pacemaker activity during aggregation in Dictyostelium
discoideum. Dev. Biol.

[9] Durston AJ, Cohen MH, Drage DJ, Potel MJ, Robertson A, Wonio D (1976). Periodic movements of Dictyostelium discoideum
sorocarps. Dev. Biol.

[10] Durston A, Vork F, Weinberger C, Vassileva-Popova JG, and Jensen EV (1978). The control of later
morphogenesis by chemotactic signals in Dictyostelium. Biochemical
and Biophysical Information Transfer in Recognition.

[11] Durston AJ, Vork F (1979). A cinematographical study of the development
of vitally stained Dictyostelium discoideum. J. Cell.
Sci.

[12] Durston AJ, Jansen HJ, In der Rieden P, Hooiveld MH (2011). Hox collinearity - a new perspective. Int. J. Dev. Biol.

[13] Durston AJ (2012). Global posterior prevalence is unique to vertebrates: a dance to the music of time?. Dev Dyn.

[14] Gingle AR, Robertson A (1979). Responses of the early chick embryo to external cAMP sources. J. Embryol. Exp. Morphol.

[15] Goodwin BC, Cohen MH (1969). A phase-shift model for the spatial and
temporal organization of developing systems. J. Theor. Biol.

[16] Lauschke VM, Tsiairis CD, Francois P, Aulehla A (2013). Scaling of embryonic patterning based on phase-gradient encoding. Nature.

[17] Matsukuma S, and Durston A (1979). Chemotactic cell sorting in Dictyostelium
discoideum. J. Embryol. Exp. Morphol.

[18] McDonald SA, Durston AJ (1984). The cell cycle and sorting behaviour in Dictyostelium iscoideum. J. Cell. Sci.

[19] Nanjundiah V (1974). A differential chemotactic response of
slime mould amoebae to regions of the early amphibian
embryo. Exp. Cell. Res.

[20] Shaffer BM (1975). Secretion of cyclic AMP induced by cyclic AMP in the cellular slime mould *Dictyostelium discoideum*. Nature.

[21] Siegert F, Weijer CJ (1992). Three-dimensional scroll waves organize Dictyostelium slugs. Proc. Nati. Acad. Sci USA.

[22] Steinbock O, Siegert F, Muller S, Weijer C (1993). Three-dimensional waves of excitation during Dictyostelium morphogenesis. Proc.
Natl. Acad. Sci. USA.

[23] Stern CD, Goodwin BC (1977). Waves and periodic events during primitive streak formation in the chick. J. Embryol. Exp. Morphol.

[24] Weijer CJ, Duschl G, David CN (1984). Dependence of cell-type
proportioning and sorting on cell cycle phase in Dictyostelium
discoideum. J. Cell. Sci.

[25] Cornelis JW, Sue AM, Antony JD (1984). A
frequency difference in optical-density oscillations of early Dictyostelium
discoideum density classes and its implications for development. Differentiation.

[26] Winfree AT (1972). Spiral waves of chemical activity. Science.

[27] Winfree AT (1973). Scroll-Shaped Waves of Chemical Activity in Three Dimensions. Science.

[28] Vasiev B, Weijer CJ (1999). Modeling Chemotactic Cell Sorting during
*Dictyostelium* discoideum Mound Formation. Biophys. J.

[29] Bretschneider T, Vasiev B, Weijer C (1999). A model for Dictyostelium
slug movement. J. Theor. Biol.

[30] Bakhtier V, Weijer J (2003). Modelling of Dictyostelium discoideum slug migration. J. Theor. Biol.

[31] Cornelis JW (1999). Morphogenetic cell movement in Dictyostelium Seminars in Cell. Dev. Biol.

[32] Bretschneider TR, Florian SF, Weijer CJ (1995). Three-dimensional scroll waves of cAMP could direct cell movement and gene expression in Dictyostelium slugs. Proc. Natl. Acad. Sci. USA.

